# Analysis of the correlation between BMI and respiratory tract microbiota in acute exacerbation of COPD

**DOI:** 10.3389/fcimb.2023.1161203

**Published:** 2023-04-25

**Authors:** Yang Cao, Xiaolin Chen, Lei Shu, Lei Shi, Mingjing Wu, Xueli Wang, Kaili Deng, Jing Wei, Jiaxin Yan, Ganzhu Feng

**Affiliations:** ^1^ Department of Respiratory Medicine, Sir Run Run Hospital, Nanjing Medical University, Nanjing, Jiangsu, China; ^2^ Department of Respiratory Medicine, The Second Affiliated Hospital of Nanjing Medical University, Nanjing, Jiangsu, China

**Keywords:** AECOPD, BMI, 16S rRNA gene sequencing, respiratory microbiota, αdiversity, PCoA

## Abstract

**Objective:**

To investigate the distribution differences in the respiratory tract microbiota of AECOPD patients in different BMI groups and explore its guiding value for treatment.

**Methods:**

Sputum samples of thirty-eight AECOPD patients were collected. The patients were divided into low, normal and high BMI group. The sputum microbiota was sequenced by 16S rRNA detection technology, and the distribution of sputum microbiota was compared. Rarefaction curve, α-diversity, principal coordinate analysis (PCoA) and measurement of sputum microbiota abundance in each group were performed and analyzed by bioinformatics methods.

**Results:**

1. The rarefaction curve in each BMI group reached a plateau. No significant differences were observed in the OTU total number or α-diversity index of microbiota in each group. PCoA showed significant differences in the distance matrix of sputum microbiota between the three groups, which was calculated by the Binary Jaccard and the Bray Curtis algorithm. 2. At the phylum level, most of the microbiota were *Proteobacteria*, *Bacteroidetes Firmicutes*, *Actinobacteria*, and *Fusobacteria*. At the genus level, most were *Streptococcus*, *Prevotella*, *Haemophilus*, *Neisseria* and *Bacteroides.* 3. At the phylum level, the abundance of *Proteobacteria* in the low group was significantly higher than that in normal and high BMI groups, the abundances of *Firmicutes* in the low and normal groups were significantly lower than that in high BMI groups. At the genus level, the abundance of *Haemophilus* in the low group was significantly higher than that in high BMI group, and the abundances of *Streptococcus* in the low and normal BMI groups were significantly lower than that in the high BMI group.

**Conclusions:**

1. The sputum microbiota of AECOPD patients in different BMI groups covered almost all microbiota, and BMI had no significant association with total number of respiratory tract microbiota or α-diversity in AECOPD patients. However, there was a significant difference in the PCoA between different BMI groups. 2. The microbiota structure of AECOPD patients differed in different BMI groups. Gram-negative bacteria (G^-^) in the respiratory tract of patients predominated in the low BMI group, while gram-positive bacteria (G^+^) predominated in the high BMI group.

## Introduction

COPD is a chronic respiratory disease characterized by persistent and progressive airflow limitation, which causes more than 3 million deaths worldwide per year (Rabe and Watz, 2017) and is estimated to become the third leading cause of death worldwide by 2030 (Fazleen and Wilkinson, 2020). Colonization and infection with respiratory pathogenic bacteria are considered to be important causes of acute exacerbation of COPD and a decline in lung function (Sethi, 2000; Leung et al., 2017). In recent years, population surveys in nine Asia-Pacific regions found that 46% of COPD patients had at least one exacerbation in the previous year, among which 19% required hospitalization. On average, 0.06 to 0.78 acute exacerbations occur per person per year (Ko et al., 2016), and 50-70% of acute exacerbations and disease progression are related to bacterial infection (Boixeda et al., 2015). The more frequent the acute exacerbations, the worse the prognosis (Wedzicha et al., 2013), and exacerbations have cumulative effects on the decline of lung function (Erhabor et al., 2021). However, classical culture techniques can only isolate a few pathogenic bacteria in AECOPD (Dickson et al., 2013), and only 10-20% of species can be detected (Bathoorn et al., 2017). The 16S rRNA gene is widely present in all prokaryotic microorganisms, containing 10 conserved regions and 9 variable regions (V1-V9). Variable region V1-V3 amplification identifies almost all pathogens, and V4-V5 amplification has a higher specificity of detection (Mendez et al., 2019). 16S rRNA sequencing can identify and detect all bacteria in clinical practice, which is not possible with traditional culture technologies (Bador et al., 2020), and a large number of short nucleotide sequences can be read in a very short time using high-throughput technologies, such as Illumina sequencing and 454 pyrosequencing (Lee et al., 2016; Allali et al., 2017). In addition, the cost of 16S rRNA sequencing is decreasing with the development of sequencing technology. Therefore, 16S rRNA gene sequencing technology can replace sputum culture as an important method for microbial detection due to its outstanding advantages of high accuracy, high sensitivity, high throughput and low cost.

Previous studies on respiratory tract microbiota in COPD have rarely been reported. In 2010, Hilty et al (Hilty et al., 2010; Moffatt and Cookson, 2017). conducted 16S rRNA gene sequencing and bacterial community analysis of oral pharynx, nasopharynx and left upper pulmonary bronchial mucosal brush samples from healthy people, asthma patients and COPD patients and found that the complex microbial community of the lower respiratory tract exists not only in patients suffering from respiratory diseases but also in healthy people. In the last decade, scholars have used sequencing technology to study the respiratory tract microbiota of COPD patients and found that in the overall microbiome, the most abundant bacteria belonged to 4 main phyla, *Proteobacteria*, *Firmicutes*, *Bacteroidetes* and *Actinobacteria*, as well as 7 main genera, *Streptococcus, Haemophilus, Moraxella, Prevotella, Acinetobacter, Neisseria* and *Fusobacterium (* Cabrera-Rubio et al., 2012; Wang et al., 2016; Yang et al., 2022). Wang et al (Lee et al., 2016; Wang et al., 2016; Wang et al., 2019; Sun et al., 2020). examined the correlation between COPD risk factors, lung function grades and respiratory tract microbiota distribution and found that smoking status, the frequency of acute exacerbations and the Global Initiative for Obstructive Lung Disease (GOLD) grade of lung function were closely related to the diversity and abundance of respiratory microbiota, including changes in α-diversity and differences in the abundance of *Proteobacteria*, *Firmicutes* as well as *Haemophilus*, *Moraxella*, *Streptococcus*, *Staphylococcus*, and *Veillonella*.

Although smoking has long been considered to be the most important risk factor for COPD, approximately 25-45% of COPD patients have no smoking history, which indicates that other factors may play important roles in the occurrence and development of COPD (Mannino and Buist, 2007; Salvi and Barnes, 2009; Barczok, 2019). Recent clinical studies have shown that low BMI is an important independent risk factor for poor prognosis in COPD patients (Schols et al., 1998). It is well known that overweight or obesity is closely related to the disease process and prognosis of diabetes, hypertension, coronary heart disease and cerebrovascular stroke (Kinlen et al., 2018). In contrast, overweight or obesity is largely protective against acute exacerbation and even prognosis in subjects suffering from COPD to a large extent (Spelta et al., 2018). Zapatero et al. (2013) conducted a retrospective study of 310,000 hospitalized COPD patients and found that obesity significantly reduced the readmission rate of acute exacerbation and the risk of death during hospitalization. Wei et al (Wei et al., 2017; Sun et al., 2019). emphasized that low BMI increases the frequency of exacerbations partly and accelerates the decline of FEV_1_ in COPD patients. Obviously, BMI is an important risk factor for COPD progression. Findings indicate that respiratory tract pathogen infection is the key factor leading to AECOPD, so it is meaningful to explore the correlation between BMI and respiratory tract microbiota in AECOPD patients.

Few studies on COPD microbiome have reported the correlation between BMI and microbiota distribution. Here, we performed a prospective 16S rRNA-based microbiome survey on sputum samples collected from patients with AECOPD of different BMI groups, sequenced the number and species of operational taxonomic units (OTUs) of the respiratory tract in each group by 16S rRNA detection sequencing technology, and analyzed the diversity and abundance of bacteria by bioinformatics methods. In this article, we explored the relationship between BMI and the distribution of respiratory tract microbiota in AECOPD, and to provide a reference for the clinical diagnosis and treatment of AECOPD patients with different BMIs.

## Methods

### Subjects and samples

Sputum samples of 38 patients with AECOPD were collected from the Department of Respiratory and Critical Care Medicine, Sir Run Run Shaw Hospital of Nanjing Medical University and Second Affiliated Hospital of Nanjing Medical University from October 2020 to June 2022, and clinical data, including sex, age, height, weight, smoking status and frequency of acute exacerbation in the previous year, were collected.

Inclusion criteria: All patients met the Global Initiative for Chronic Obstructive Lung Disease (GOLD) diagnostic criteria for COPD and were in an acute exacerbation. Hospitalization was required for related treatment. Exclusion criteria: There was a medical history of severe pulmonary diseases or combined with other chronic respiratory diseases such as bronchial asthma, cystic fibrosis, severe bronchiectasis and other structural pulmonary diseases. There was a history of diabetes or autoimmune diseases. Suffering from malignant tumor within 5 years. There was a history of acute exacerbation of COPD or pulmonary infection as well as antibiotics use within 3 months before the study.

The patients were divided into three groups according to BMI (kg/m^2^), low BMI (13 patients, BMI ≤ 18.5 kg/m^2^), normal BMI (13 patients, BMI 18.5-23.9 kg/m^2^) and high BMI (12 patients, BMI≥24.0 kg/m^2^) (Zhu et al., 2020). All groups underwent Brilliance iCT and spirometry to exclude other chronic respiratory diseases, such as bronchial asthma, cystic fibrosis, severe bronchiectasis and other structural lung disease, and to confirm the presence of airway obstruction. All subjects met the condition of forced expiratory volume in the first second/forced vital capacity (FEV1/FVC) <70% and the predicted percentage of FEV _1_ (FEV _1_%) <80% (Celli and Macnee, 2004).This study was approved by the Medical Ethics Committee of the Sir Run Run Shaw Hospital of Nanjing Medical University (2021-SR-020).

Sputum (2-3 ml) was collected from each subject and stored at −80°C until further use before antibiotics and other treatments on the day after admission. 16S rRNA gene amplicon sequencing and analysis were conducted by OE Biotech Co., Ltd. (Shanghai, China).

### DNA exacerbation and PCR amplification

Bacterial DNA was isolated from the sputum using a MagPure Soil DNA LQ Kit (Magen, Guangdong, China) following the manufacturer’s instructions. DNA concentration and integrity were measured by a NanoDrop 2000 spectrophotometer (Thermo Fisher Scientific, Waltham, MA, USA) and by agarose gel electrophoresis, respectively. The genomic DNA was used as a template for PCR amplification with barcoded primers and Tks Gflex DNA Polymerase (Takara). For bacterial diversity analysis, the V3-V4 variable regions of 16S rRNA genes were amplified with universal primer pairs (343F: 5′-TACGGRAGGCAGCAG-3′; 798R: 5′-AGGGTATCTAATCCT-3′). The reverse primer contained a sample barcode, and both primers were tagged with an Illumina sequencing adapter.

### Library construction and sequencing

The amplicon quality was visualized using gel electrophoresis. The PCR products were purified with Agencourt AMPure XP beads (Agencourt) and quantified using a Qubit dsDNA assay kit. The concentrations were then adjusted for sequencing. Sequencing was performed on an Illumina NovaSeq6000 with two paired-end read cycles of 250 bases each (Illumina Inc., San Diego, CA; OE Biotech Company; Shanghai, China).

### Bioinformatics analysis

Raw sequencing data were in FASTQ format(All raw data has been uploaded to NCBI database:PRJNA934046). Paired-end reads were then preprocessed using Trimmomatic software to detect and cut off ambiguous bases (N). Low-quality sequences with average quality scores below 20 were also cut off using a sliding window trimming approach. After trimming, paired-end reads were assembled using FLASH software. The parameters of assembly were 10 bp of minimal overlapping, 200 bp of maximum overlapping and 20% of maximum mismatch rate. Sequences were further denoised as follows: reads with ambiguous sequences or homologous sequences and reads of less than 200 bp were abandoned. Reads with 75% of bases above Q20 were retained. Then, reads with chimaeras were detected and removed. These two steps were achieved using QIIME software (version 1.8.0).

Clean reads were subjected to primer sequence removal and clustering to generate operational taxonomic units (OTUs) using Vsearch software with a 97% similarity cut-off. The representative read of each OTU was selected using the QIIME package. All representative reads were annotated and blasted against Silva database Version 138 (www.arb-silva.de) using RDP classifier with a 70% confidence threshold (Yilmaz et al., 2014).

### Biodiversity and community similarity analyses

The absolute and relative abundance of the sequence number of each OTU in each sputum sample were calculated using R language, and the OTUs of each sample were ranked according to the abundance value at the phylum and genus levels. α-Diversity analysis was used to assess microbiota diversity within the group, including Shannon, Simpson, Chao1 and Coverage indices. The β-Diversity analysis was used to assess the similarity and difference in microbiota between different BMI groups and was followed by principal coordinates analysis (PCoA). Binary Jaccard and Bray Curtis algorithms were used to calculate the distance matrix of each group, and multivariate variance analysis (Adonis) was used to determine significant differences in microbiota between different BMI groups.

### Statistical analysis

SPSS 25.0 and GraphPad Prism 9.0 were used for data analysis. Fisher’s exact test was used to compare the sex distribution between groups. Age, FEV1%pred and FEV1/FVC satisfied asymptotic normality and were compared by ANOVA. BMI, frequency of acute exacerbation, absolute abundances of bacteria, the Shannon, Simpson, Observed Species, and Coverage indices of α-diversity did not meet asymptotic normality, so Kruskal-Wallis nonparametric (K-W) test was used to calculate *P* values for them between groups. The absolute abundances of bacteria were presented as M(IQR), representing the median (interquartile range). After K-W test was used to calculate the differences of bacteria between different BMI groups, rank-converted was performed for the distribution among groups, and ANOVA was used to calculate the *P* value. *P <*0.05 indicates that the difference is statistically significant.

## Results

### Subjects

The clinical characteristics of the 38 patients enrolled in this study are presented in **Table 1**. All subjects ranged in age from 61 to 88 years, with an average age of 72.3 years, including 33 males and 5 females. According to BMI, the patients were divided into low BMI (13 subjects), normal BMI (13 subjects) and high BMI (13 subjects) groups. There were no significant differences in sex, age, FEV1/FVC, EFV1%, BMI or frequency of acute exacerbation.

**Table 1:** Comparison of general clinical data of patients in different BMI groups.

	low BMI group	normal BMI group	high BMI group	*P* value
Gender (M:F)	11/2	12/1	10/2	0.85 5^a^
BMI (kg/m2)	17.73 (0.81)	21.66 (1.70)	25.95 (3.05)	<0.001^b^
Age	76.69 ± 4.31	72.85 ± 7.20	76.33 ± 5.76	0.201^c^
FEV1/FVC	52.54 ± 2.80	56.25 ± 3.06	56.83 ± 2.29	0.495^c^
FEV1%	49.46 ± 3.44	57.11 ± 5.38	59.89 ± 2.36	0.176^c^
Frequency	2.31 (1.00)	1.38 (1.00)	1.75 (1.75)	0.015^b^

a Fisher’s exact test.

b Kruskal-Wallis test.

c Analysis of variance (ANOVA).

### Rarefaction curve of sputum microbiota

**Figure 1:**
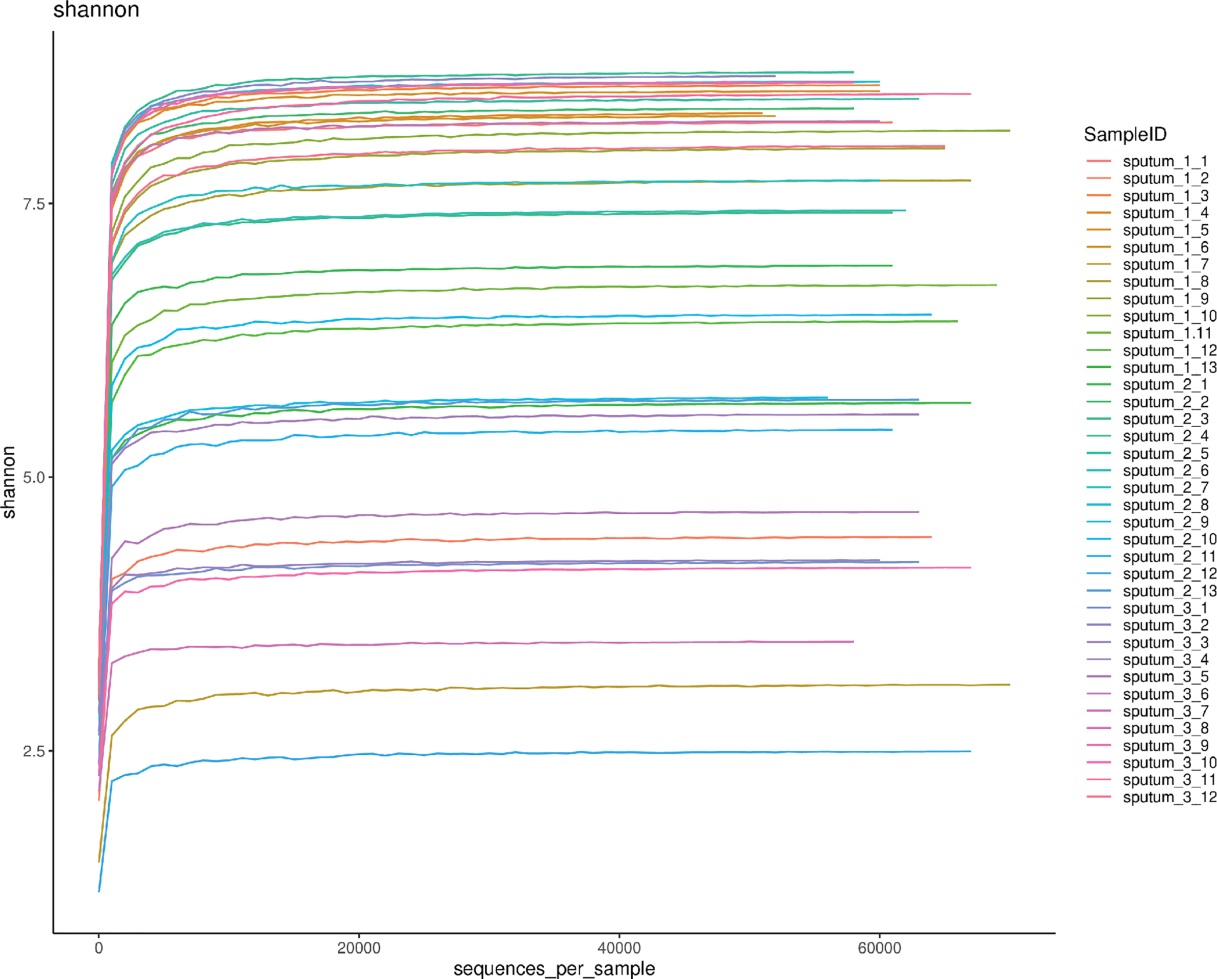
The rarefaction curve of 38 respiratory tract microbiota. Shannon-Wiener analysis of 38 respiratory samples. The x-coordinate is the number of sequences per sample, and the y-coordinate is the Shannon rarefaction measure. The higher the y value is, the higher the community α-diversity is.

A total of 2367303 usable 16S rRNA sequences were obtained from all samples by Illumina sequencing, with an average of 62297 sequences per sample. Then, 13067 OTUs were delineated at a 97% similarity level after quality decontamination and filtering. Rarefaction curves of α-diversity were obtained while increasing the number of sequences (**Figure 1**).

### Profiles of sputum microbiota OTUs

**Figure 2:**
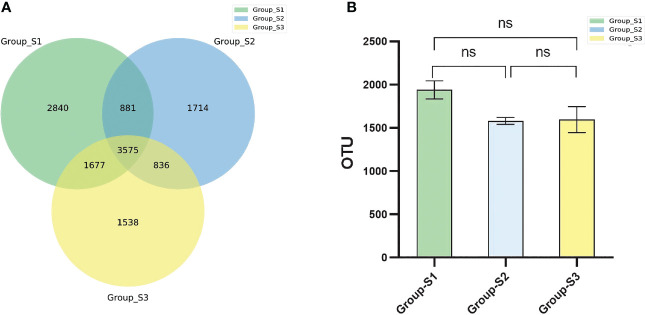
Comparison of bacterial OTU numbers in different groups. **(A)** Venn diagram of the OTUs in the respiratory tract specimens of the three groups. **(B)** Boxplots of the OTU numbers in respiratory tract specimens among the three BMI groups. (ANOVA, ns: *P*<0.05, there were no statistical differences between different groups.).

The sputum microbiota OTUs of each BMI group were calculated. There were 8973 OTUs in the low BMI samples, 7006 OTUs in the normal BMI samples and 8507 in the high BMI samples, and 3575 OTUs existed in the three groups simultaneously. There was no statistically significant difference in OTU numbers between groups using analysis of variance (ANOVA) for comparison (**Figure 2**).

**Figure 3:**
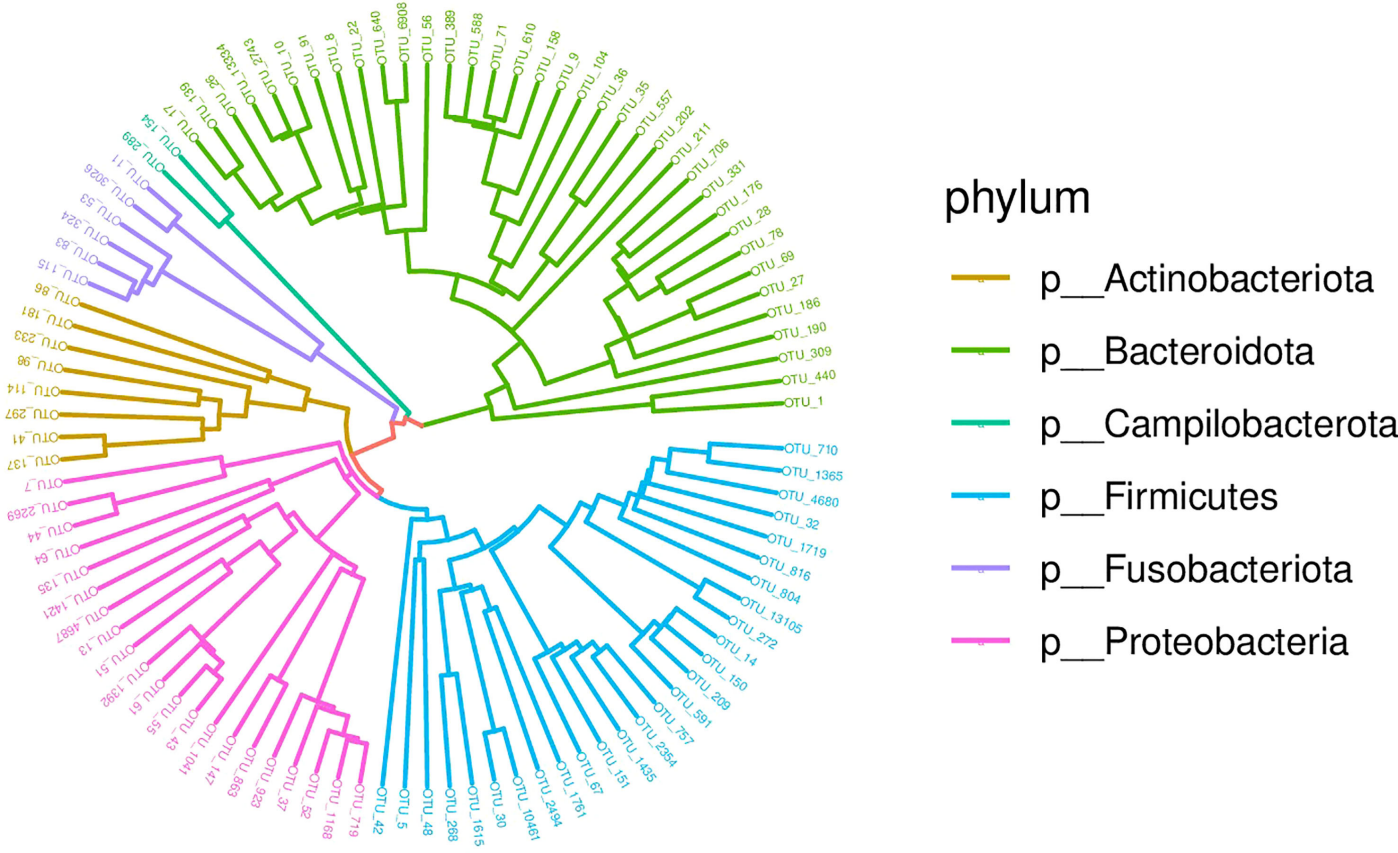
Phylogenetic tree diagram of the top 50 OTUs. Phylogeny studies the formation and evolution history of species. In this Circle Tree, the top 50 species with the highest abundance during evolution are clustered into six phyla.

**Figure 4:**
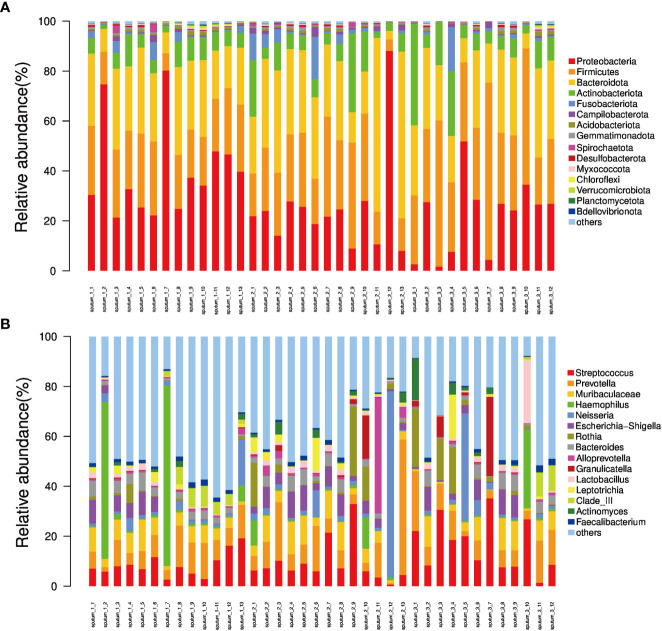
The relative abundance of phyla and genera derived from sequencing of each sputum sample. **(A)** Bar plot of relative abundance in each sample at the phylum level; **(B)** Bar plot of relative abundance in each sample at the genus level.

Afterwards, the abundance of each OTU was counted using R language, and the most abundant OTUs were selected to construct an evolutionary tree, among which *Actinobacteria*, *Bacteroidetes*, *Campilobacterota*, *Fusobacteria*, *Firmicutes* and *Proteobacteria* had the highest abundance (**Figure 3**). Based on the overall bacterial phyla composition, the most abundant bacteria belonged to one of five bacterial phyla: *Proteobacteria* (30.0%), *Bacteroidetes* (27.5%), *Firmicutes* (27.5%), *Actinobacteria* (9.5%), and *Fusobacteria* (2.6%). At the genus level, the most abundant bacteria were *Streptococcus* (11.3%), *Prevotella* (10.3%), *Haemophilus* (5.7%), *Neisseria* (5.4%), and *Bacteroides* (3.2%), all of which are typical members of the lung microbiota. *Rothia, Escherichia-Shigella* and *Muribaculaceae* were also common (**Figure 4**).

### Microbial diversity of sputum microbiota in different BMI groups

**Figure 5:**
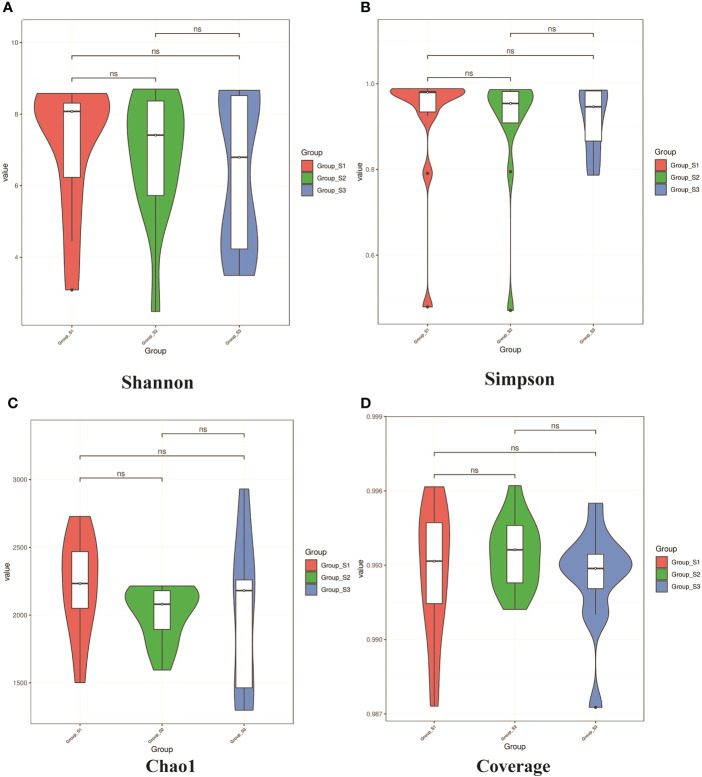
Violin diagrams of α-diversity on Shannon, Simpson index, Chao I and Coverage indices from each BMI group. **(A, B)** Violin diagrams of microbiota on Shannon and Simpson indices respectively, representing the diversity of microbiota within the group. **(C)** Violin diagram of microbiota on Chao1 indice, representing the richness of microbiota within the group. **(D)** Violin diagram of microbiota on Goods coverage indice, representing the sequencing depth of microbiota within the group.(K-W test, ns: *P*<0.05, there were no statistical differences between different groups.).

**Figure 6:**
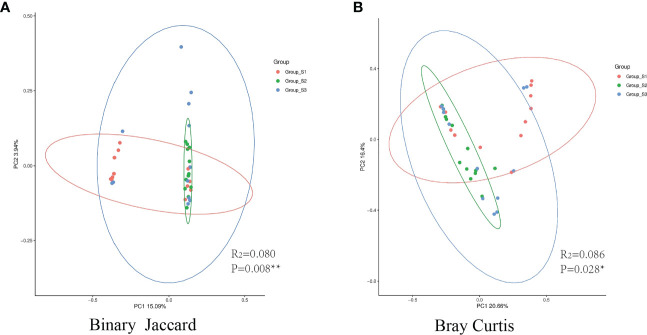
PCoA between different BMI groups. **(A)** The binary Jaccard algorithm was used to calculate the distance matrix of each group, and Adonis was used to analyze the difference between the presence or absence of species. **(B)** The Bray Curtis algorithm was used to analyze the difference in microbiota abundance. (The circle represents the 95% confidence interval of each group. Points farther away indicate greater differences by visualization). (*:*P*<0.05, there was a significant difference between the three groups in the distance matrix; **:*P*<0.01, there was a great significant difference between the three groups.).

The Shannon, Simpson, Chao I and Coverage indices were calculated to evaluate the α-diversity of bacteria in different BMI groups. There was no significant difference in the α-diversity of sputum bacteria between the three groups (*P*>0.05,**Figure 5**). Adonis analysis was used for PCoA based on the Binary Jaccard and Bray-Curtis algorithms, and the results showed significant differences in respiratory tract microbiota between the BMI groups (R_2_ = 0.080, *P*=0.008; R2 = 0.087, *P*=0.028, **Figure 6**).

### Abundance difference of main sputum microbiota OTUs in different BMI groups

The absolute abundance of the main OTUs in different BMI groups was compared. The Kruskal-Wallis test was used to calculate the differences of bacteria in different BMI groups, after the distribution, pairwise comparison was rank-converted, and ANOVA was used to calculate the *P* value.

**Figure 7:**
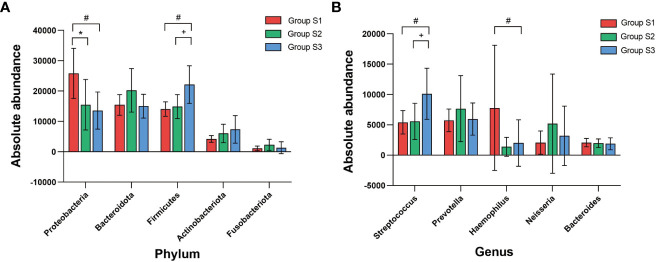
Correlation analysis between BMI and absolute abundance of core bacterial OTUs **(A)** Comparison of the absolute abundance of core OTUs at the phylum level between BMI groups. **(B)** Comparison of the absolute abundance of core OTUs at the genus level between BMI groups. (K-W test, **^#^**:*P*<0.05, there was a significant difference between S3 and S1 in absolute abundance; *:*P*<0.05, there was a significant difference between S2 and S1 in absolute abundance;**^+^**:*P*<0.05, there was a significant difference between S3 and S2 in absolute abundance).

At the phylum level, the absolute abundances of *Proteobacteria* in the low, normal and high BMI groups were 23938 (15870), 13940 (6972) and 15133 (14700), respectively, and there were significant differences between the different groups (low BMI group vs. normal BMI group, *P*=0.031 and low BMI group vs. high BMI group, *P*=0.023). The absolute abundances of *Firmicutes* in the low, normal and high BMI groups were 14625 (4927), 15566 (11220.5) and 17530.5 (14885.75), respectively, and significant differences were found between the low BMI group and the high BMI group (*P*=0.012) as well as between the normal BMI group and the high BMI group (*P*=0.030). At the genus level, the absolute abundances of *Haemophilus* in the low, normal and high BMI groups were 581 (2487), 320 (624) and 280.5 (513.25), respectively, and the difference between the low BMI group and the high BMI group was significant (*P*=0.013). The absolute abundance of *Streptococcus* in the low, normal and high BMI groups were 4405 (3106.5), 3847 (2760.5) and 8971.5 (12534), respectively, and the absolute abundance of *Streptococcus* in high BMI group was significantly higher than that in the low BMI group (*P*=0.049) and normal BMI group (*P*=0.014). There was no significant difference in the abundance of other OTUs between BMI groups (**Figure 7**).

## Discussion

COPD is a chronic pulmonary disease characterized by irreversible airflow limitation. With the progression of the disease, complications, such as pulmonary heart disease and respiratory failure, may occur, which significantly affects the quality of life of patients and greatly increases the economic burden of disease treatment (Toraldo and Conte, 2019). The drugs currently used to treat COPD mainly include β2 receptor agonists, M3 receptor blockers and glucocorticoids (Viniol and Vogelmeier, 2018). In recent years, great progress has been made in the half-lives, dosage forms, administration methods, devices, etc. of these drugs, which has greatly improved patient symptom relief (Fujimoto, 2014). Despite of these advancements, FEV1%pred and FEV1/FVC are still decreasing progressively in COPD patients (Tantucci and Modina, 2012). Studies have demonstrated that recurrent airway infection plays a key role in alveolar structure destruction and airway remodelling in COPD patients (Wang et al., 2018). Therefore, effective reduction or control of respiratory tract infection is crucial to improve the disease course and even prognosis of COPD patients. The respiratory tract is an open airway in which microorganisms from the surrounding environment coexist, colonize or infect when immune function declines. Obviously, it is of great significance to carry out relevant studies on the respiratory microbiome of COPD patients in different states to understand the occurrence, development, prevention and control of the disease. Studies have shown that BMI affects the diversity and abundance of intestinal microbiota (Stanislawski et al., 2019). Other studies have demonstrated bidirectional regulation between the intestinal microbiota and respiratory microbiota, which is a famous theory of the “intestinal-lung axis” proposed in recent years (He et al., 2017). Therefore, BMI may affect the respiratory tract microbiota more or less. In recent years, with the rapid development of sequencing technology, the conditions for carrying out respiratory tract microbiome research have become increasingly mature. In this study, we used 16S rRNA sequencing technology to study the differences and correlations of microbiome distribution in AECOPD with different BMIs. 16S rRNA sequencing technology uses next-generation sequencing technology (NGS) of 454 pyrophosphate or Illumina to sequence millions of gene fragments (Kozich et al., 2013). Some sequences of bacterial ribosomal RNA (rRNA) can be used to identify the genetic differences of microorganisms, and rRNA can be divided into three types according to the sedimentation coefficient, 5S, 16S and 23S. The 5S rRNA gene has a short sequence and little genetic information, and the 23S rRNA gene has a long sequence and a high base mutation rate. Only the 16S rRNA sequence is suitable in length and copy number for species identification by sequencing; it not only runs well in sequencing technology platforms but also reflects the distribution difference of microbiota. The V3-V5 variable region of 16S rRNA is highly specific and can reflect the characteristics of microbial community components through PCR amplification, high-throughput sequencing and bioinformatics analysis (Holm et al., 2019). In this study, OE Biotech Co., Ltd, which we collaborated with, performed 16S rRNA genetic testing using the next-generation sequencing method of llumina. Compared with the traditional PCR method, it intercepts more specific sequences for analysis. Compared with the first-generation sequencing methods such as Sanger, large-scale parallel sequencing can be carried out simultaneously, instead of only sequencing one DNA fragment. Compared with the whole genome shotgun sequencing (WGS) method, it has a longer sequence number and is more attractive in terms of cost-effectiveness. Compared with metagenome sequencing, it has obvious advantages in run time and sequencing cost (Ranjan et al., 2016).

In this study, a total of 38 AECOPD patients were enrolled and divided into a low BMI group, normal BMI group and high BMI group. Previous studies showed that diabetes and autoimmune diseases such as systemic lupus erythmatosus, rheumatoid arthritis,etc. are related to the microbiota disorder in human intestine and other organs (Xu et al., 2021).Zhang et al. indicated that microbiota-host interactions are important in regulating the malignant transformation of cancer cells and cancer-related immunity (Zhang et al., 2022). And Budden showed that chronic respiratory diseases such as asthma, cystic fibrosis and bronchiectasis were associated with changes in microbial diversity or abundance (Budden et al., 2019).Therefore, in our research, we excluded patients who combined with diabetes, autoimmune diseases, tumors and structural lung diseases. To avoid an effect of antibiotics on prokaryotic 16S rRNA results, we excluded patients who received any antibiotics within three months when we selected enrollees. And to date, the effects of other drug such as ICS therapy on the airway microbiome in COPD are unclear, some scholars believe that ICS lead to a relative reduction in α-diversity (Leitao et al., 2021), other studies showed that there was no significant differences in bacterial α diversity index with or without ICS inhaled in chronic lung disease (Huang et al., 2022). Therefore, we temporarily ignored the influence of inhaled drugs on the conclusion. In the follow-up study, we can expand the sample and carry out a systematic study. Subjects in this study were selected from the respiratory and critical care departments of two local teaching hospitals, Sir Run Run Shaw Hospital of Nanjing Medical University and Second Affiliated Hospital of Nanjing Medical University. There was no significant difference in general clinical data, such as sex and age, indicating that the sample selection in this study was random. There was no significant difference in FEV1%pred or FEV1/FVC of AECOPD patients in the different BMI groups, but the annual frequency of acute exacerbation in the low BMI group was significantly higher than that in the normal BMI group.

Airway samples were obtained from spontaneous or induced sputum of AECOPD patients. To avoid oral background bacteria, such as *Firmicutes*, *Actinomyces* and *Spirochaetes (* Verma et al., 2018), we asked patients to gargle with warm water and H_2_O_2_ repeatedly before taking samples, which could reduce oral colonization bacterial contamination to a large extent and ensure the reliability of respiratory tract microbiota detection. The literature has demonstrated that the microbiota composition of bronchoalveolar lavage fluid (BALF) is very similar to that of bronchial mucosa and can represent the structure of respiratory tract microbiota (Cabrera-Rubio et al., 2012). Some studies have also suggested that after repeated oral cleaning to remove the influence of colonized bacteria in the oral cavity, sputum can represent the respiratory microbiota to some extent (Holz et al., 2000). Indeed, sputum is more susceptible to upper respiratory tract microbiota contamination than BALF, and there are certain limitations in selecting sputum rather than BALF for studies of the respiratory microbiota. In fact, most patients with AECOPD were breathless and even complicated with heart failure and other diseases. Invasive examinations, such as bronchoscopy and alveolar lavage, had risks of hypoxia, airway spasm and arrhythmia, which reduced the safety of the operation and affected the collection of samples (Melo-Dias et al., 2022). Therefore, in this study, sputum samples were used instead of BALF to study the distribution of respiratory tract microbiota.

16S rRNA detection technology was used to analyze sputum samples, and a large number of sequences were obtained. The number of sequences continuously increased until the rarefaction curve approached the plateau, indicating that the number of sequences was sufficient to cover most of the OTUs. Although some additional sequences may generate some new OTUs, the index of α-diversity would not change significantly. OTU abundance obtained by sputum sequencing showed that *Proteobacteria*, *Bacteroidetes*, *Firmicutes*, *Actinobacteria*, *Fusobacteria* and *Prevotella, Streptococcus, Neisseria, Haemophilus, Bacteroidetes* were the main OTUs of COPD patients, and the most dominant phylum identified in the sputum samples was *Proteobacteria*, accounting for 30% of the microbiome, which was basically consistent with the report of Leiten et al (Leiten et al., 2020; Su et al., 2022). No significant differences were found in the total OTU quantity or α-diversity of sputum in the different BMI groups, indicating that the BMI of AECOPD patients does not affect the quantity of respiratory tract microbiota; the microbiota were shown to be rich and even in all groups. PCoA of the microbiota in different BMI groups showed that the presence or absence of bacterial species and their species abundance distance matrix were significantly different, suggesting that the BMI of AECOPD patients was correlated with the species and abundance of respiratory microbiota. The binary Jaccard and Bray Curtis algorithms are the two distance matrix calculation algorithms of PCoA. The former represents the difference between the presence or absence of species, while the latter not only represents the difference in the presence or absence of species but also the difference in microbiota abundance. Combined application of the two methods can fully reflect the differences in species and abundance between groups.

Next, we found differences in the microbiota abundance in different BMI groups by analyzing the correlation between BMI and microbiota abundance in each group. *Proteobacteria* abundance was significantly increased in the low BMI group compared to the high and normal BMI groups, especially for the genus *Haemophilus*. *Firmicutes* abundance was significantly higher in the high BMI group than in the low BMI group and normal BMI group, with *Streptococcus* being dominant. *Proteobacteria* is an important phylum, and all Proteobacteria are gram-negative (G^-^). The bacterial cell membrane has an outer and an inner membrane. The peptidoglycan wall is thin and is coated by lipopolysaccharide (LPS) biofilms (Sommer et al., 2013; Supuran et al., 2013). The common clinical bacteria genera in this phylum mainly include *Haemophilus*, *Moraxella*, *Pseudomonas*, *Enterobacter*, *Neisseria* and other pathogenic bacterial groups (Guan et al., 2018). A large number of studies have shown that G^-^ bacteria can induce an inflammatory response through a series of virulence factors, such as LPS and lipooligosaccharide (LOS), leading to vascular endothelial injury, tissue necrosis and even multiple organ failure (Bassetti and Shorr, 2018). The reasons for low BMI in COPD patients may be multifaceted, including their own genetic factors and chronic gastroesophageal diseases (such as chronic gastritis, oesophageal reflux disease, etc.) and the course of COPD itself, which is also a critical factor. With the prolongation of the course of the disease, the lung function of COPD patients progressively declines, resulting in an increased breathing rate to meet the body’s demand for oxygen during activities and even at rest, thereby increasing the work of breathing muscles and reducing patient BMI. According to the clinical data of this study, we noticed that although there were no significant differences in the lung function indices FEV_1_% and FEV1/FVC between the low BMI group and the normal and high BMI groups, there was a downwards trend, which reflected the increase in respiratory muscle work in the low BMI group to a certain extent. In addition, the results of this study show that the average annual frequency of acute exacerbations in the low BMI group was significantly higher than that in the normal BMI group, which is consistent with other studies (Steriade et al., 2019). Although the mechanism of the increased frequency of acute exacerbations in patients with low BMI is not very clear, most of them are considered to be related to the decline in lung function, and a severe decline in lung function is associated with more obvious remodelling of airway structure, which provides an anatomical basis for the colonization or infection of Proteobacteria (Yilmaz et al., 2021). Therefore, in clinical practice, the possibility of G^-^ bacterial infection should be given high consideration in the diagnosis of AECOPD patients with low BMI at the initial stage of admission. It is recommended that empiric antibiotic treatment covering *Proteobacteria* should be administered prior to obtaining the results of respiratory aetiology tests. *Firmicutes* are mostly gram-positive (G^+^), and only a few pathogens, such as *Mollicutes* (e.g., *Mycoplasma*), cannot be stained by the Gram method due to their lack of a cell wall. *Firmicutes* have a monolayer cell wall structure surrounded by a thick peptidoglycan layer and include pathogenic bacterial groups, such as *Streptococcus* and *Staphylococcus*, as well as probiotics, such as *Lactobacillus* and *Lactococcus (* Megrian et al., 2020; Padayachee et al., 2020; Taib et al., 2020). Studies on metabolic diseases have found that the *Firmicutes*/*Bacteroidetes* ratio plays an important role in maintaining the balance of the normal intestinal environment, and an increase or decrease in the ratio can lead to intestinal ecological disorders. Obesity is positively correlated with the presence of *Firmicutes* in the intestinal tract (Stojanov et al., 2020). In this study,the results showed that compared with the low BMI group, the *Firmicutes* detection level in the respiratory tract in the normal and high BMI groups was significantly higher, which suggested the existence of crosstalk between the intestine and lung, namely, the gut-lung axis. What is the exact reason for the increase in *Firmicutes* in the respiratory tract of patients with a high BMI? Do the *Firmicutes* bacteria come from the intestinal tract? This remains to be studied.

This study has the following limitations:

1. The sample size of each group included in the study was relatively low, which made the study prone to bias to a certain extent.
2. Most of the included patients had poor lung function and could not undergo BALF sampling after bronchoscopy. Sputum was used to replace BALF for the study, which may be disturbed by oropharyngeal microbiota to some extent.

## Data availability statement

The data presented in the study are deposited in the NCBI repository, accession number: BioProject: PRJNA934046.

## Ethics statement

The studies involving human participants were reviewed and approved by Medical Ethics Committee of the Sir Run Run Hospital of Nanjing Medical University(2021-SR-020). The patients/participants provided their written informed consent to participate in this study.

## Author contributions

YC, XC, and LShu conceived and designed the study and take responsibility for the integrity of the data and the accuracy of the data analysis. LShi, MW, KD, JW, XW, and JY assisted in data collection, extraction, and evaluation of the eligibility of the original data. YC and LShu analyzed the data. YC, XC, and GF interpreted the data and contributed to the writing of the final version of the manuscript. All authors contributed to the article and approved the submitted version.
